# Acupuncture and Acupoints for Managing Pediatric Cerebral Palsy: A Meta-Analysis of Randomized Controlled Trials

**DOI:** 10.3390/healthcare12171780

**Published:** 2024-09-05

**Authors:** Ya-Yun Cheng, Ying-Yu Huang, Tsung-Hsien Yang, Yi-Jung Chang, Ren-Huei Fu, Hsing-Yu Chen

**Affiliations:** 1 Division of Chinese Acupuncture and Traumatology, Center of Traditional Chinese Medicine, Chang Gung Memorial Hospital, Taoyuan 333008, Taiwan; b0105018@cgmh.org.tw; 2 Division of Chinese Internal and Pediatric Medicine, Center of Traditional Chinese Medicine, Chang Gung Memorial Hospital, Taoyuan 333008, Taiwan; b0105030@cgmh.org.tw (Y.-Y.H.); 8905001@cgmh.org.tw (T.-H.Y.); 3 School of Traditional Chinese Medicine, College of Medicine, Chang Gung University, Taoyuan 333008, Taiwan; 4 Department of Pediatrics, Chang Gung Memorial Hospital, Chang Gung University College of Medicine, Taoyuan 333008, Taiwan; r64321@cgmh.org.tw; 5 Department of Pediatrics and Neonatology, Chang Gung Memorial Hospital, Linkou 333423, Taiwan; rkenny@cgmh.org.tw

**Keywords:** acupuncture, pediatric, cerebral palsy, meta-analysis, core acupoints

## Abstract

Background: Acupuncture is frequently used to manage pediatric cerebral palsy (CP), yet updated evidence is needed to guide future research and clinical practice. Methods: Seven databases were searched from 1994 to 26 June 2023. Randomized controlled trials (RCTs) involving body, scalp, or ear acupuncture for managing CP, excluding acupoint injection, catgut embedding, electro-acupuncture, or laser acupuncture, were included. Results: Twenty RCTs with 1797 participants were analyzed. Acupuncture groups had better improvements in gross motor function measure (GMFM) scores by 5% (mean difference: 5.93, 95% CI: 3.67–8.19, *p* < 0.001, I^2^ = 57%); a 16% higher probability to yield prominent improvement in effectiveness rate (ER) (risk ratio: 1.16, 95% CI: 1.08–1.25, *p* < 0.001, I^2^ = 0%); and better outcomes in the Modified Ashworth Scale (MAS) (standardized mean difference [SMD]: 0.3, 95%, CI: 0.11–0.49, *p* < 0.001, I^2^ = 0%), the Berg Balance Scale (BBS) (SMD: 2.48; 95% CI: 2.00–2.97, *p* < 0.001, I^2^ = 72%) and ADL (SMD: 1.66; 95% CI: 1.23–2.08, *p* < 0.001, I^2^ = 91%). Studies with eight core acupoints identified from all ninety-five acupoints had better ER. Conclusions: Acupuncture, especially using core acupoints, may be effective for managing symptoms in children with CP.

## 1. Introduction

Cerebral palsy (CP) is a neurological condition that affects motor control and muscle coordination. It is typically caused by brain damage during early development, impacting an individual’s ability to move and maintain balance from childhood onward. Recent evidence indicated that CP occurs in two to three out of one thousand live births [1]. According to recent studies, CP presents itself in various forms, including spastic diplegia (35%), spastic quadriplegia (20%), dyskinetic (15%), ataxic (5%), hemiplegic (30%), and mixed (15.4%) type [2]. Each type presents unique challenges, affecting muscle tone and mobility differently. The most common clinical features are movement disorders and associated disabilities, such as balance issues and sensory deficits [3]. Consequently, long-term complications that may arise, including pain (75%), intellectual disability (50%), inability to walk (33%), hip displacement (33%), inability to talk (25%), epilepsy (25%), behavior disorders (25%), bladder control problems (25%), sleep disorders (20%), drooling (20%), blindness (10%), and deafness (4%) [4]. As a result, the managing strategy for CP primarily focuses on improving motor function, relieving disabilities, and reducing the risk of further complications [1]. Physical and occupational therapies are conventionally used for pediatric patients, including exercise interventions [3]. However, some studies question the efficacy of exercise intervention in improving gross motor function (GMF) [5,6]. Other approaches included a combination of speech therapy, medication, and new approaches such as robotic assistance [7,8] or aquatic therapy [9]. However, these new treatment strategies have not yet demonstrated superior outcomes compared to conventional managements. Additionally, the relatively small number of relevant studies and participants is a concern [10].

In traditional Chinese medicine (TCM), CP is not specifically documented. Instead, it falls under the categories of “five late”, “five soft”, and “five hard”, which all describe the symptoms experienced by CP patients. According to the principles of “TCM syndrome differentiation and treatment”, the success of treatment in children is closely tied to the accuracy of syndrome differentiation. Aside from the syndrome differentiation, numerous studies indicated that acupuncture can manage the symptoms of CP. Acupuncture, a common and classic practice rooted in the principle of TCM, has been used as a feasible and complementary therapy for CP [11]. In post-stroke patients, acupuncture can help relax spastic muscles and improve joint mobility [12,13] and can also have a positive impact on neuroplasticity [14,15]. Additionally, acupuncture may benefit CP-associated comorbidities, such as drooling [16], intellectual disability [17], speech disorders [18,19], and sleep disorders [20], and may also improve digestive function [21] and reduce anxiety and depression [22]. Many clinical studies have assessed the effect of acupuncture on managing CP. Some studies indicated that combining occupational therapy with acupuncture can improve spasticity in CP and some showed that Chinese herbal medicine (CHM) combined with acupuncture yielded promising outcomes [23,24] In 2010 and 2019, Zhang et al. and Li et al. reported that acupuncture seemed effective for CP; however, the enrolled studies were outdated and different outcomes for CP were lacking [25,26]. In 2019, Zhu et al. examined the effects of acupuncture on muscle spasticity but did not conduct a further meta-analysis [27]. Recently, Kang et al. reported a meta-analysis on using Jingjin therapy, also known as needle knife therapy, for abnormal muscle tension in children with CP rather than pure acupuncture [28]. Due to the limited indication of Jingjin therapy, the paper primarily addressed muscle tension issues in CP, and other aspects of CP, such as quality of lifer and gait balance, were not reported. On the other hand, another two meta-analyses also reported the potential benefit of acupuncture for CP with other acupuncture-like therapies, including acupoint injection, latch needles, and laser acupuncture, which were not extensively used in clinical practice and may increase the heterogeneity of enrolled studies [29,30]. Furthermore, these previous meta-analyses commonly enrolled clinical trials with various acupoints in the intervention group. The complexity of the chosen acupoints posed significant challenges in interpreting the summarized effect due to the heterogeneity in study design and could not provide recommendations about choosing acupoints.

This study aimed to investigate and summarize the efficacy of pure acupuncture in managing CP. The acupoints used in clinical trials were analyzed to elucidate core acupoints for CP. The combination of efficacy evaluation and core acupoints determination makes the results of this study an important resource for both Western medicine and TCM doctors.

## 2. Materials and Methods

### 2.1. Eligibility Criteria

Inclusion criteria of this study were as follows:

1. Randomized controlled trials (RCTs);
2. A diagnosis of CP, confirmed by a physician’s evaluation based on established diagnostic criteria;
3. An intervention consisting of body, scalp, or ear acupuncture;
4. A control group that did not receive any acupuncture treatment or other TCM treatments;
5. No restrictions based on age, ethnicity, or language.

Exclusion criteria consisted of the following:

1. Non-human studies;
2. Studies not published in peer-reviewed journals;
3. Studies that did not have a control group;
4. Studies that did not provide age and gender in the results;
5. Studies that included interventions such as acupoint injection, catgut embedding, electro-acupuncture, or laser acupuncture to avoid the possible bias in results estimation and interpretation.

### 2.2. Information Sources and Search Strategy

An extensive literature search was conducted across several databases, including PubMed, Cochrane Library, Scopus, EMBASE, ClinicalTrials.gov, PubMed Central, and the China National Knowledge Infrastructure (CNKI), from 1994 to 26 June 2023. We used MESH terms to search for relevant information, including keywords such as “cerebral palsy”, “acupuncture”, “acupuncture therapy”, “acupuncture, ear”, “acupuncture points”, “scalp acupuncture”, and “randomized controlled trial”, while excluding “animal”. “The same keywords with corresponding words in Chinese were used for searching clinical trials written in Chinese on CNKI. Detailed search strategies are listed in Supplementary Table S1. The study protocol was registered in PROSPERO prior to data collection (No. CRD42023427334).

### 2.3. Data Extraction

The data extraction process was carried out independently by Ya-Yun Cheng, Ying-Yu Huang, and Tsung-Hsien Yang using a predefined data collection form. In cases with unclear eligibility, discussions involving Hsing-Yu Chen were conducted to resolve them. The extracted data included key elements such as publication year, country, study design, study setting, diagnosis criteria, sample size, and the gender distribution and age of participants. Collected treatment details for both the intervention and control groups encompassed specific treatment methods, control methods, the use of sham acupuncture, the frequency and duration of treatments, and measurement of outcomes. Additionally, if deemed necessary by the reviewing author, efforts were made to contact the corresponding authors of the clinical studies to address any missing or incomplete data.

### 2.4. Quality Assessment

The evaluation of methodological rigor was conducted by Ya-Yun Cheng and Ying-Yu Huang, employing a well-established assessment tool: the Cochrane Collaboration’s risk-of-bias (RoB) [31]. Any disagreements were resolved through constructive discussions involving the project directors, Professor Hsing-Yu Chen and Tsung-Hsien Yang.

### 2.5. Outcome Measures

Values of gross motor function measure (GMFM) [32] were set as the primary outcome with baseline and end-of-study changes extracted. Additionally, effectiveness rate (ER) for overall improvement, Modified Ashworth Scale (MAS) [33] for spastic motor function evaluation, Activities of Daily Living (ADL) [34], and Berg Balance Scale (BBS) [35] for gait and balance were extracted at baseline and at the end of the study as the secondary outcomes. These assessment methods were commonly used to evaluate progress in motor function for CP patients. The ER was assessed by the investigators of each enrolled clinical trials and was commonly defined as the percentage of participants who achieved “prominent improvement” (i.e., disease severity improving by more than 50% in terms of symptom relief), with a higher ER indicating more effective intervention.

### 2.6. Statistical Analysis

Data were analyzed using Cochrane Review Manager 5.4 (Cochrane, London, UK). Mean values with standard deviation were used to presented numerical data, while proportions were used to demonstrate categorical data. Also, we calculated the risk ratio with a 95% confidence interval (CI) for studies that used the ER as an outcome measurement. For studies that used numerical scale as outcomes, we calculated the standardized mean difference (SMD) and mean difference (MD) with a 95% CI. A random effect model (Mantel–Haenszel for dichotomous data and inverse variance for continuous data) was used to model the pooled data. Study heterogeneity was assessed using the I^2^-statistic. We derived weight from the number of cases and the effect size of each study, and then the results are pooled based on these weights.

To evaluate the improvement of GMFM, we conducted a pooled analysis based on the percentage of GMFM improvement. Additionally, network analysis was employed to identify core acupoints by visually illustrating the combinations of acupoints commonly utilized for CP. Network analysis is a data-mining technique used to explore the most significant components in a complex network of connected parts [36,37,38,39]. This technique has previously been used to distinguish the effect of core CHM treatment on specific diseases from a large number of prescriptions with multiple herbs [36,37,40]. Since all studies used multiple acupoints in their respective acupuncture groups, we employed network analysis to build an acupoint network for CP and determine the core acupoints in the enrolled studies, using a similar approach to that used for CHM. For this reason, acupoints with high prevalence and frequent connections with other acupoints were considered core acupoints.

Furthermore, subgroups analysis was conducted to assess effectiveness across various treatment durations and among the identified core acupoints. The studies with the highest heterogeneity were excluded in sensitivity tests to examine the data consistency. The entire analysis was performed using Cochrane’s review manager (version 5.4), and the NodeXL (https://www.smrfoundation.org/nodexl/ (accessed on 3 May 2024)) was used to analyze the acupoints network. Results at *p* ≤ 0.05 were deemed statistically significant. To confirm the effect of acupuncture with better controls for type I and type II errors, a trial sequential analysis (TSA) was carried out with a preset of 5% type I error and 90% power [41,42]. Additionally, a funnel plot was used to assess the possible publication bias.

## 3. Results

### 3.1. Search Results

**Figure 1:**
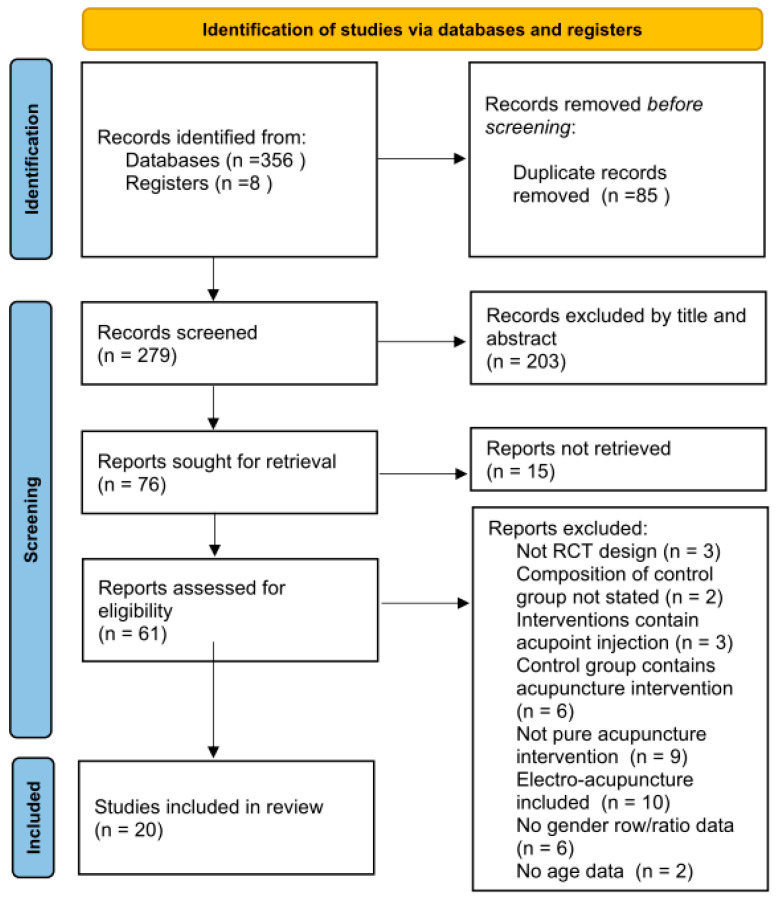
Flow chart.

Figure 1 shows the flowchart of the study design. The initial phase of our research involved both electronic and manual searches, which collectively yielded 364 articles that had been published up to 26 June 2023. A thorough examination of titles and abstracts led to the exclusion of 288 studies, primarily due to duplications or missing/unmatched titles or abstracts. Following this initial screening process, we retrieved and closely scrutinized the full texts of 76 references. Finally, a comprehensive content evaluation based on the aim of this study led to the inclusion of 20 studies, encompassing a total of 1797 participants (Figure 1).

### 3.2. Description of the Included Studies

#### 3.2.1. Characteristics of Studies

Table 1 provides detailed information of the studies enrolled at the final stage. All of the included studies were randomized controlled trials evaluating the efficacy of pure acupuncture for CP, employing two parallel treatments. However, three of these were three-arm controlled trials; in these cases, only data comparing acupuncture and control groups were extracted in these cases. All the selected studies were conducted in China. The frequency of interventions varies across studies, ranging from 8 to 27 weeks, with treatment sessions occurring from one to seven times a week. A total of seventy-nine acupoints were used in the enrolled studies, ranging from one and twenty-nine acupoints.

#### 3.2.2. Participant Characteristics

The age of the participants varied, ranging from 1.6 ± 0.25 years old to 12.4 ± 6.2 years old. The number of male participants (*n* = 1087, 63.2%) exceeded female participants (*n* = 633, 36.8%). Seventeen papers were written in Mandarin Chinese and three in English. Studies were conducted from 2008 to 2022, with nine published recently (2017–2022). Sixteen studies adhered to the China National Clinical Diagnosis and Classification Criteria for CP. Among these, six studies used the Rehabilitation Guideline for Cerebral Palsy in China [62] for diagnostic and classification, while ten studies followed the Guideline for Cerebral Palsy in China [63]. Four of the enrolled studies did not explicitly specify the criteria, but one of them noted the use of the GESELL score. Forty percent (8/20) of the studies enrolled spastic participants, and the rest of the studies did not specify the subtype of CP.

#### 3.2.3. Design of Control Group Interventions

In most of the reviewed studies, the primary management in the control arms mainly consisted of standard CP management, including physical and/or occupational therapies. However, it is noteworthy that two studies deviated from this pattern by employing MyoTrac bio-stimulation as their principal intervention. Myo Trac is a rehabilitation device that provides stimulation to the patient’s affected limbs, aiding inactive movement performance. Additional speech therapy was utilized in five of the enrolled studies. Four studies included massage in conjunction with standard management in the control group. Regarding massage, two articles briefly mentioned its conclusion but did not detail the methods used [48,60]. In contrast, two other articles described specific pressing techniques employed [44,46]. Both approaches involve manual pressure on acupoints, though the methods used in these articles differ due to the complexity of massage techniques. Other interventions observed in individual studies included balance training, hyperbaric oxygen therapy (existing still strong controversies in legitimating this therapy in case of CP), and intelligence therapy. Balance training involved placing the patient on an unbalanced shaking board or big gymnastic ball and then letting them to return to the neutral place independently. Intelligence motor therapy utilized technology and competitive games to train limbs movements. Finally, two of the studies included sham acupuncture as a secondary control group.

#### 3.2.4. Design of Intervention Group Interventions

The enrolled studies utilized two kinds of acupuncture, separated by the locations of acupoints: scalp acupuncture, which exclusively targeted scalp acupoints, and body acupuncture, which focused solely on body acupoints. Additionally, three studies (15%) adopted a comprehensive approach by employing both types. Among these, six studies (30%) primarily utilized scalp acupuncture, while body acupuncture was used in eleven studies (55%).

#### 3.2.5. Types of Outcome Assessments

Twelve (60%) of the studies used a GMFM assessment, six (30%) used an ER, six (30%) employed MAS, three (15%) utilized ADL, and two (10%) employed the BBS. Other assessments used in two studies are detailed Table 1.

### 3.3. Quality of the Enrolled Studies

**Figure 2:**
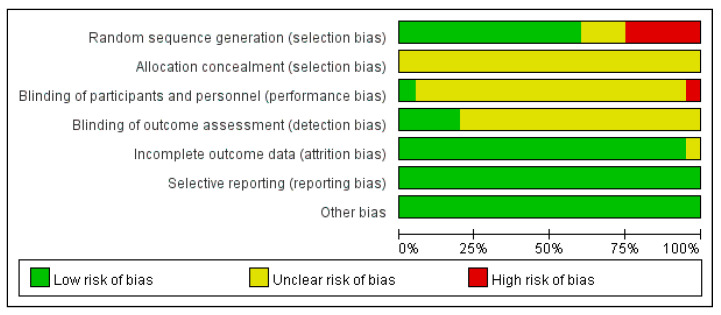
Methodological quality assessment of 20 included studies by using Cochrane Collaboration’s risk-of-bias (ROB) assessment tool: risk of bias graph.

**Figure 3:**
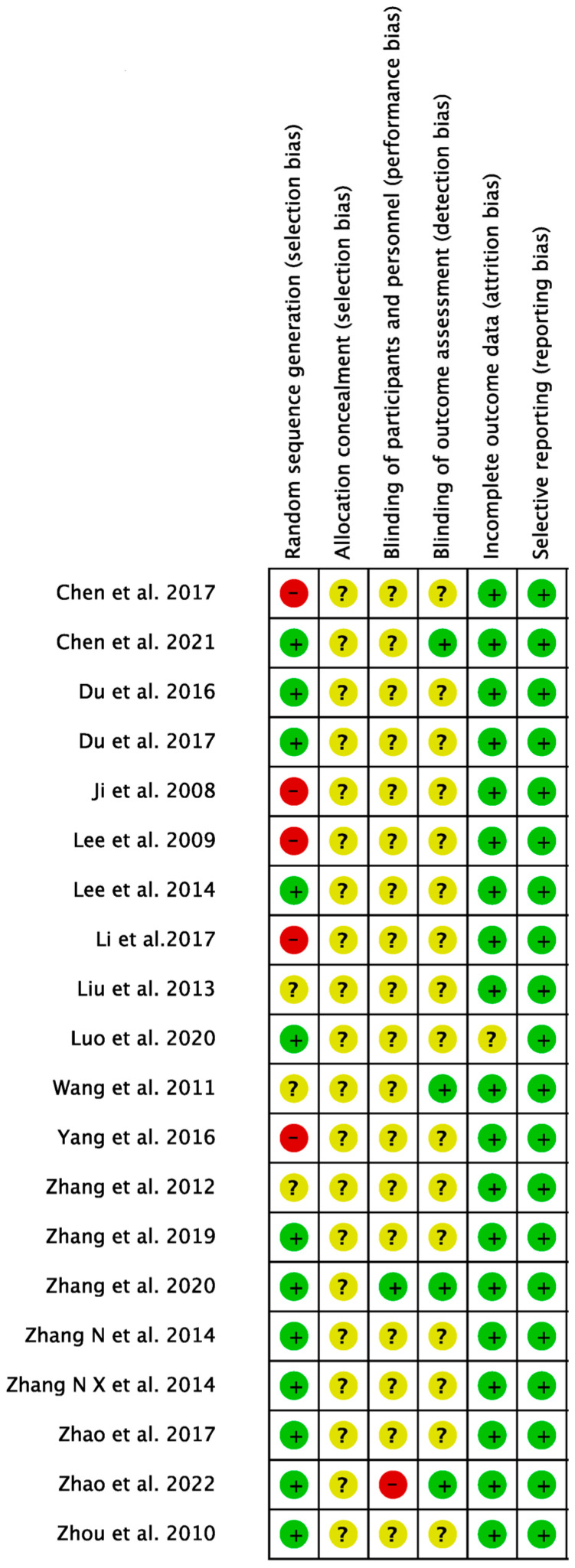
Risk of bias summary. Red circles represent a high risk of bias, yellow circles denote an unclear risk of bias, and green circles indicate a low risk of bias [23,43,44,45,46,47,48,49,50,51,52,53,54,55,56,57,58,59,61,64].

Based on the RoB assessment (Figure 2 and Figure 3), most results indicated an unclear status regarding selection bias, performance bias, and detection bias. A low-risk status represented by the clinical trial demonstrated the methods to eliminate these biases. An unclear status arose when the articles did not provide detailed information on experimental methods and procedures. Conversely, a high-risk status was attributed to significant errors in experimental methods or not address these issues in the content. Notably, five studies were identified as having a high risk of selection bias (25%), while twelve studies (60%) were found to have a low risk of bias in this aspect.

Most of the articles with a low-risk of selection bias mentioned using random number methods or the use of machines for random group allocation at least. In contrast, articles with high bias risk, such as those by Li et al. [46] and Lee et al. [59], used the parity of the last digit of patient numbers for grouping. Chen et al. [58] used a lottery system based on patient numbers, while Yang et al. [61] and Ji et al. [54] used appointment times for grouping. Among all articles favoring acupuncture in GMFM, Lee et al. [59] and Ji et al. [54] had a high risk of selection bias, and others had low risks of bias for studies with ER as outcome assessments, such as Ji et al. In terms of detection bias, four studies (20%) demonstrated low risk. Furthermore, all studies consistently showed low risk of bias in attrition and reporting.

### 3.4. Meta-Analysis of the Included Studies

#### 3.4.1. Improvement in Motor Function

**Figure 4:**
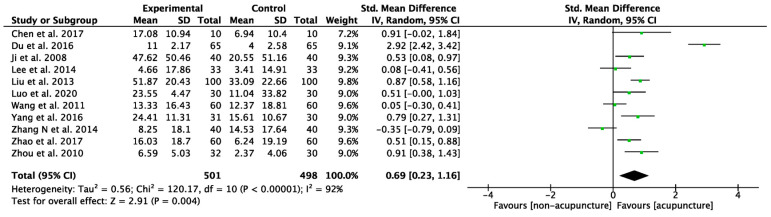
Meta-analysis of improvement for gross motor function measure (GMFM) The green label represents the standardized mean difference calculated for each individual study. The diamond symbol represents the overall standardized mean difference and its 95% confidence interval, calculated from all studies combined [45,48,50,51,52,53,54,56,58,61,64].

**Figure 5:**
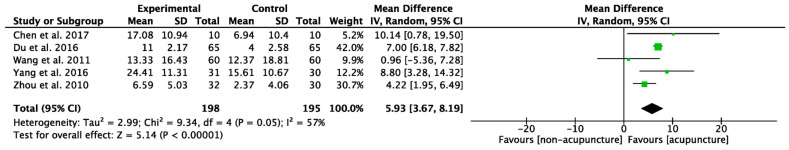
Meta-analysis of improvement for gross motor function measure (GMFM), extracted for evaluating the improvement of GMFM percentage The green label represents the standardized mean difference calculated for each individual study. The diamond symbol represents the overall standardized mean difference and its 95% confidence interval, calculated from all studies combined [48,52,53,58,61].

We used GMFM to assess changes in gross motor function among children diagnosed with CP, with higher scores indicating better motor function. The pooled analysis of GMFM was based on 11 studies (Figure 4). One study was excluded due to unusable raw data. The mean scores for intervention groups ranged from 4.66 to 51.87, while for control groups they ranged from 2.37 to 33.09. Consequently, the acupuncture groups showed a significantly greater improvement compared to the control groups, with a moderate SMD (SMD: 0.69, 95% CI: 0.23–1.16, *p* = 0.004). However, significant heterogeneity was observed across the studies in GMFM assessment (I^2^ = 92%, *p* = 0.004). On average, there was a 5% improvement in GMFM scores (MD: 5.93, 95% CI: 3.67–8.19, *p* < 0.001, I^2^ = 57%, Figure 5).

#### 3.4.2. Prominent Improvement Based on ER

**Figure 6:**
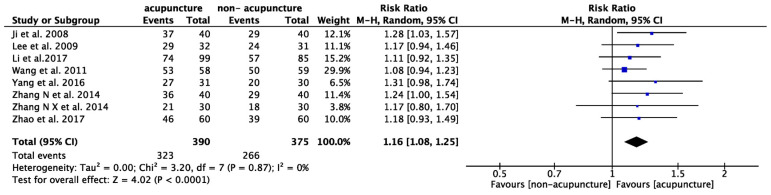
Meta-analysis of effective rate (ER). The blue label represents the risk ratio for each individual study. The diamond symbol represents the overall risk ratio and its 95% confidence interval, calculated from all studies combined [45,46,49,50,52,54,59,61].

In eight studies that included ER, acupuncture was associated with a 16% higher probability of achieving prominent improvement compared to control groups (RR: 1.16, 95% CI: 1.08–1.25, *p* < 0.001, I^2^ = 0%; Figure 6). A higher risk ratio (RR) indicates better overall symptom relief. For ER, TSA showed a consistent result with a sufficient cumulative number of cases to achieve the preset power (Supplementary Figure S1).

#### 3.4.3. Spastic Motor Function

**Figure 7:**
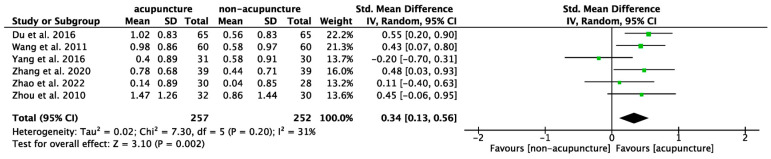
Meta-analysis of improvement for Modified Ashworth Scale (MAS) The green label represents the standardized mean difference calculated for each individual study. The diamond symbol represents the overall standardized mean difference and its 95% confidence interval, calculated from all studies combined [23,48,52,53,55,61].

In six studies that included spastic motor function, the average incremental score ranged from 0.14 to 1.47 in the intervention groups and from 0.04 to 0.86 in the control groups, demonstrating greater improvement in MAS with acupuncture treatment (SMD: 0.34; 95% CI: 0.13–0.56, *p* = 0.002, I^2^ = 31%; Figure 7).

#### 3.4.4. Balance Function

**Figure 8:**
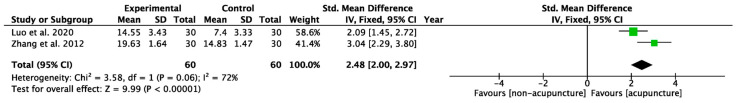
Meta-analysis of improvement for Berg Balance Scale (BBS) The green label represents the standardized mean difference calculated for each individual study. The diamond symbol represents the overall standardized mean difference and its 95% confidence interval, calculated from all studies combined [56,57].

In two studies that included balance function, the average score in the acupuncture groups ranged from 14.55 to 19.63, whereas the mean score in control groups ranged from 7.4 to 14.83. This indicated greater efficacy with acupuncture treatment (SMD: 2.48; 95% CI: 2.00–2.97, *p* < 0.001, I^2^ = 72%; Figure 8).

#### 3.4.5. Daily Activity Function

**Figure 9:**
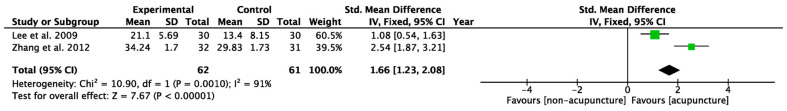
Meta-analysis of improvement for Activity of Daily Living (ADL) The green label represents the standardized mean difference calculated for each individual study. The diamond symbol represents the overall standardized mean difference and its 95% confidence interval, calculated from all studies combined [57,59].

In two studies that included daily activity function, the mean score in the intervention groups ranged from 21.1 to 34.24, whereas the mean score of the control groups ranged from 13.4 to 29.83. This indicates that ADL scores improved under acupuncture treatments (SMD: 1.66; 95% CI: 1.23–2.08, *p* < 0.001, I^2^ = 91%; Figure 9).

### 3.5. Network Analysis for Core Acupoints Exploration

**Figure 10:**
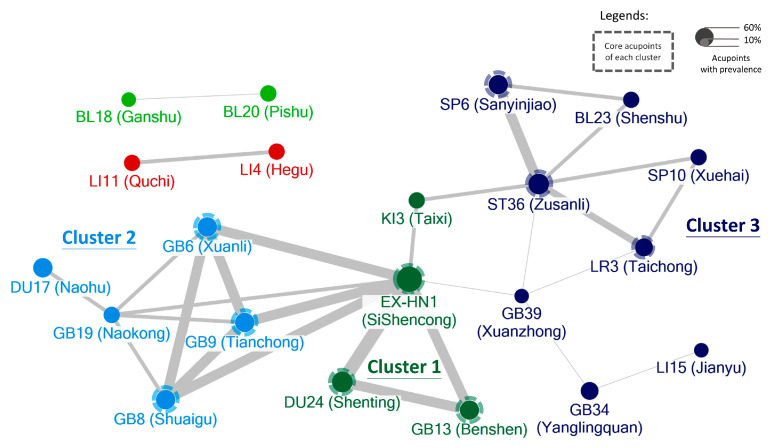
The network analysis of core acupoints for cerebral palsy was derived from trials included in this study. The displayed points represent acupoints collected from these trials. Different colors represent different acupoint clusters. The size of each node corresponds to its prevalence, with larger nodes indicating higher prevalence. Acupoints with same colors are regarded as commonly used in combination. The most prevalence acupoint are placed in the center of the cluster, namely EX-HN1 (Sishencong), GB6 (Xuanli), and ST36 (Zusanli). The width of connecting lines indicates the prevalence of the combination of two acupoints, with greater line width suggests more substantial and frequent co-administration of these acupoints in the network. The figure was created using the software NodeXL.

The acupoints used for the network analysis in the selected articles are listed in Supplementary Table S2. Three core acupoints combinations were identified (Figure 10). The first set of core acupoints included EX-HN1 (Sishencong), which had strong connections to DU24 (Shenting) and GB13 (Benshen) (cluster 1). The second set (cluster 2) comprised GB6 (Xuanli), GB8 (Shuaigu), and GB9 (Tianchong). The third, smaller set (cluster 3) included ST36 (Zusanli) along with LR3 (Taichong) and SP6 (Sanyinjiao).

### 3.6. Subgroup Analysis: Studies with Different Duration and Core Acupoints

**Figure 11:**
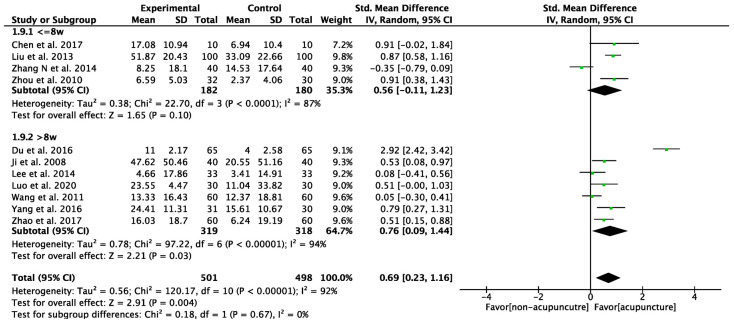
Subgroup analysis of different treatment durations on GMFM The green label represents the standardized mean difference calculated for each individual study. The diamond symbol represents the overall standardized mean difference and its 95% confidence interval, calculated from all studies combined [45,48,50,51,52,53,54,56,58,61,64].

A subgroup analysis was conducted by dividing the treatment group into two distinct categories based on treatment duration: those receiving treatment for less than 8 weeks and those receiving it for more than 8 weeks. The 8-week cut-off was chosen because most studies (35%) use an 8-week course in treatment group, and, additionally, an 8-week duration is a common time point in clinical practice for assessing efficacy. The group with the longer treatment duration (SMD: 0.76; 95% CI: 0.09–1.44, *p* = 0.03, I^2^ = 94%; Figure 11) showed a notable improvement in GMFM compared to the group with the shorter treatment duration.

**Figure 12:**
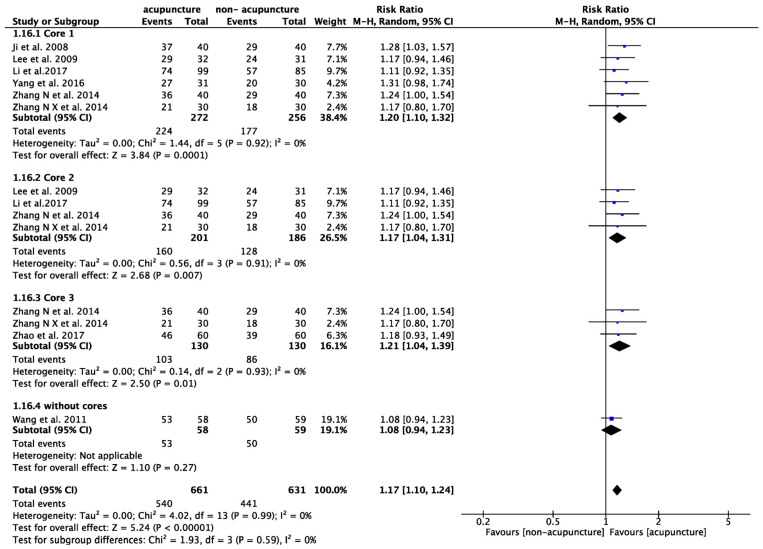
Meta-analysis of the rate to achieve prominent improvement in effective rate (ER) within different core acupoints The blue label represents the risk ratio for each individual study. The diamond symbol represents the overall risk ratio and its 95% confidence interval, calculated from all studies combined [45,46,49,50,52,54,59,61].

Subgroup analysis of core acupoints showed that studies utilizing core acupoints had a higher proportion of prominent improvement in ER compared to those without core acupoints. Cluster 1 had a 20% higher probability of achieving prominent improvement compared to the control group (RR: 1.20, 95% CI: 1.10–1.32, *p* < 0.001, I^2^ = 0%, Figure 12). Cluster 2 had a 17% higher probability (RR: 1.17, 95% CI: 1.04–1.31, *p* = 0.007, I^2^ = 0%, Figure 12), and Cluster 3 had a 21% higher probability (RR: 1.21, 95% CI: 1.04–1.39, *p* = 0.01, I^2^ = 0%, Figure 12). In contrast, studies without the core acupoints did not achieve prominent improvement compared to control groups (RR: 1.08, 95% CI: 0.94–1.23, *p* = 0.27, Figure 12).

### 3.7. Sensitivity Analysis

**Figure 13:**
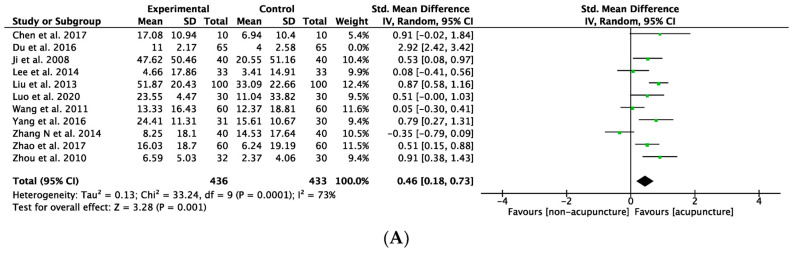

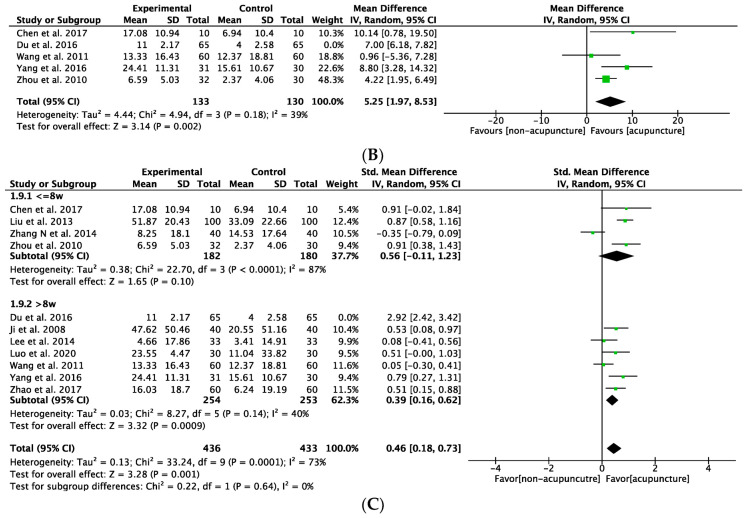
Sensitivity tests. (**A**) Meta-analysis of improvement for gross motor function measure (GMFM), excluding Du et al. (2016) [45]. (**B**) Meta-analysis of percentage improvement for gross motor function measure (GMFM), excluding Du et al. (2016). (**C**) Subgroup analysis of different treatment durations, excluding Du et al. (2016). The green label represents the standardized mean difference calculated for each individual study. The diamond symbol represents the overall standardized mean difference and its 95% confidence interval, calculated from all studies combined [45,48,50,51,52,53,54,56,58,61,64].

Among the 11 included studies of GMFM improvement, Du et al. (2016) [48] reported results with the highest heterogeneity. Excluding Du’s study, there was reduced heterogeneity, and the effect of acupuncture on managing CP symptoms remained consistent (SMD: 0.46, 95%CI: 0.18–0.73, *p* = 0.001, I^2^ = 73%, Figure 13A). The percentage improvement in GMFM did not change after excluding Du’s study. (MD: 5.25; 95% CI: 1.97–8.53, *p* = 0.002, I^2^ = 39%, Figure 13B) The observed improvement remained consistent regarding different treatment durations, even after excluding data from that paper (SMD: 0.39; 95% CI: 0.16–0.62, *p* < 0.001, I^2^ = 40%, Figure 13C). 

### 3.8. Adverse Events

Four studies reported adverse events during the evaluation; however, none of the included studies indicated discomfort or serious adverse events.

### 3.9. Funnel Plot

**Figure 14:**
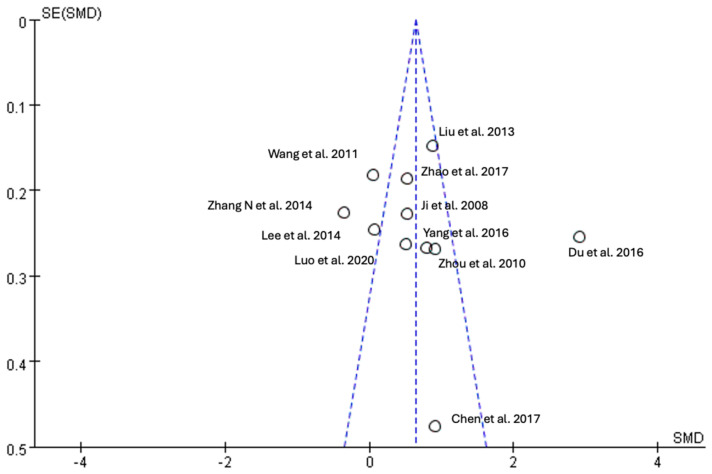
Funnel plot for potential publication bias [45,48,50,51,52,53,54,56,61,64].

The funnel plot revealed possible publication bias due to the absence of studies with small sample sizes (Figure 14).

## 4. Discussion

In contemporary society, there is a growing emphasis on seeking evidence for the benefits of practicing acupuncture. In this systemic review, 364 articles were collected and only 20 articles were enrolled. Studies were excluded because of duplication, being non-RCT, involving non-pure acupuncture interventions, or lacking essential general data. Most of the included articles did not provide much detail on blinding select; however, there was only one articles had significant flaws. This difficulty is attributed to the challenge of implementing blinding for acupuncturists.

In this meta-analysis, we analyzed twenty studies including 1797 children and adolescent participants with CP and investigated core acupoints to clarify the role of acupuncture in managing CP. Motor function improved more prominently among acupuncture participants than control groups, with higher improvement observed in GMFM scores. Even after excluding Du’s study, the results remain positive. Due to being overly optimistic, Du’s study was excluded in a sensitivity test, as its inclusion could introduce bias. Although there was decrease in the improvement of GMFM and percentage of GMFM after excluding Du’s study, the trend remained, showing a potential advantage compared to the control group. The positive result was consistent with TSA, sensitivity tests, and subgroup analysis. Moreover, acupuncture participants showed more prominent overall improvement in ER. Additionally, acupuncture participants also had better improvement in other CP-related disabilities, such as spastic, balance, or daily activity dysfunctions. As the first study to explore the most crucial acupoints for CP, we identified three sets of core acupoints: (1) EX-HN1 (Sishencong), DU24 (Shenting), and GB13 (Benshen); (2) GB6 (Xuanli), GB8 (Shuaigu), and GB9 (Tianchong); and (3) ST36 (Zusanli), LR3 (Taichong), and SP6 (Sanyinjiao). The use of these acupoints, along with longer treatment duration, was associated with better outcomes. To the best of our knowledge, this is the first study to use TSA to confirm the pooled efficacy of acupuncture and to provide information about core acupoints for CP. Acupuncture may have a positive impact on managing CP. There were no confirmed instances of serious adverse events associated with acupuncture. This meta-analysis demonstrates the feasibility of using acupuncture as a potentially advantageous alternative and complementary therapy within the framework of standard therapy for pediatric CP.

Based on these data, acupuncture therapy, when added to the standard interventions for CP patients, demonstrates significant advancements across various domains of physical and functional well-being. Notable improvements were observed in motor skills, muscle tone, balance, and overall daily functioning. Our meta-analysis indicates that acupuncture can lead to a 5% enhancement in GMFM for CP patients. A previous meta-analysis and systematic review found that acupuncture had a similar effect on GMFM as did neuromuscular electrical stimulation [65]. However, that study showed limitations in improving walking ability with neuromuscular electrical stimulation. It is possible that acupuncture therapy may overcome this obstacle as our study also shows the potential benefit in ADL and spasticity. In addition, acupuncture shows comparable functionality to Lokomat robotic rehabilitation for CP patients, particularly concerning GMFM outcomes, as used in robot-assisted gait training [66].

Moreover, acupuncture may outperform various types of robot-assisted gait training in balance rehabilitation. It was concluded that robot-assisted gait training does not significantly improve MAS and BBS scores, whereas acupuncture does and is much cheaper to implement, making it more accessible for patients from various backgrounds. However, combining different treatment modalities can result in synergistic effects. In addition to improvement in overall symptoms and motor disabilities, our research suggests that CP patients with severe (ADL below 30) to moderate dependence (ADL 35–60) could gain more benefits from acupuncture management. Previous studies have indicated that the capacity to carry out daily activities impacts quality of life [67], thereby affecting family stress in terms of both physical caregiving and emotional well-being. Importantly, these notable improvements are not restricted to a specific subtype of CP; rather, acupuncture therapy is applicable as long as the patient meets the diagnosis criteria for CP.

We also delineated three sets of core acupoints, which may represent the first choice for pediatric CP acupuncture interventions. Acupoint sets 1 and 2 were a combination of Jhih san jhen (combination of EX-HN1 [Sishencong], DU24 [Shenting], and GB13 [Benshen]) and Nie san jhen (combination of GB6 [Xuanli], GB8 [Shuaigu], and GB9 [Tianchong]), respectively. These are common acupoint sets used for brain-related diseases. For example, Jhih san jhen (combination of EX-HN1 [Sishencong], DU24 [Shenting], and GB13 [Benshen]) has been used to treat vascular dementia [68] and impaired cognition [64]. Similarly, Nie san jhen (combination of GB6 [Xuanli], GB8 [Shuaigu], and GB9 [Tianchong]) has been used to treat post-stroke aphasia [69] and spastic motor function after stroke [70]. Previous studies suggest that Jhih san jhen (combination of EX-HN1 [Sishencong], DU24 [Shenting], and GB13 [Benshen]) and Nie san jhen (combination of GB6 [Xuanli], GB8 [Shuaigu], and GB9 [Tianchong]) could substantially improve the development level and quality of daily life of children with intellectual disabilities, potentially due to their effects on regulating serum neuron-specific enolase and monoamine neurotransmitters [17]. Another core acupoint, EX-HN1 (Sishencong), has been used for treating motor function after cerebral ischemia and was found to enhance the survival and specialized development of NG2-expressing cells through the stimulation of signaling pathways induced by the brain-derived neurotrophic factor [71]. DU24 (Shenting) was able to enhance the learning and memory capabilities of rats with vascular dementia, which could be attributed to the repair of microvascular structures and the enhancement of cerebral blood flow [72]. In Chinese medicine, the flow of qi and blood and qi enhancement may symbolize the effect on neuroplasticity [14,15].

Regarding sham acupuncture, two studies included a sham acupuncture group. Sham acupuncture is important in acupuncture research as it aims to closely mimic the tactile sensation of real acupuncture [73]. However, due to the small number of studies and the varying methods of sham acupuncture used, one with minimal skin contact and the other with a Streitberger placebo needle, a subgroup meta-analysis was not conducted. Additionally, some studies [74] have noted significant variations in sham acupuncture methods, with no standardized practices established, such as differences in needle insertion depth and the use of other tools. This lacks standardization makes it difficult to compare the results collectively.

This study was subject to some limitations. First, the predominance of Chinese-language articles limits the diversity of ethnicities, with a major focus on Asians. Therefore, questions regarding the generalizability of acupuncture across different ethnicities warrant further investigation. Importantly, this meta-analysis underscores the feasibility of conducting such clinical studies in other populations. Second, most trials had small sample sizes and an unclear quality from RoB assessment, impacting the overall reliability of the research. Third, the extensive use of diverse acupoints suggests the need for larger-scale studies to establish more conclusive evidence. Fourth, the high heterogeneity observed in statistical aspects may stem from the diverse study designs. To address this, we focused solely on pure acupuncture and explored core acupoint to reduce heterogeneity. The subgroup analysis of core acupoints further demonstrated a significant reduction in heterogeneity. Therefore, we consider core acupoints crucial for guiding clinical practice and enhancing homogeneity in future clinical trials.

## 5. Conclusions

The results of this meta-analysis suggest that the combination of acupuncture and rehabilitation training constitutes an effective treatment for children diagnosed with CP. According to our results, three sets of core acupoints are potentially crucial for managing CP in clinical practice: (1) EX-HN1 (Sishencong), DU24 (Shenting), and GB13 (Benshen); (2) GB6 (Xuanli), GB8 (Shuaigu), and GB9 (Tianchong); and (3) ST36 (Zusanli), LR3 (Taichong), and SP6 (Sanyinjiao). Despite the need for cautious interpretation of our results due to several study limitations, practitioners may consider using acupuncture in conjunction with rehabilitation training as a potential treatment approach for children with CP. More extensive and larger-scale research is necessary to validate and extend these findings.

## Figures and Tables

**Table 1:** Characteristics of included studies.

Reference	Diagnostic Criteria	Drop-Out Rate	Sample Size (T)	Sample Size (C)	Sex (T, M/F)	Sex (C, M/F)	Age (T)	Age (C)	Intervention (T)	Sham Group	Intervention (C)	Frequency and Duration	Primary Outcome
[43]	CP with GESELL(DQ) < 75	-	11	9	8/3	6/3	33.6 m/o	32.8 m/o	Body/Scalp acupuncture	No	PT, OT	Three times a week for 3 months	GESELL
[44]	Spastic CP (2015 RGCPC)	-	31	31	18/13	17/14	20 ± 3 m/o	21 ± 2 m/o	Body acupuncture	No	PT, electronic biofeedback, ST, massage	Five times a week for 45 days	GESELL, GMFM-88, s-EMG
[45]	2006 GCPC	-	60	60	38/22	36/24	2.9 ± 1.0 y/o	2.8 ± 1.0 y/o	Body acupuncture	No	OT	Once a day for consecutive 20 days with a total of 3 courses, within courses with 30 days pause	GMFM, ADL, effective rate
[46]	2015 RGCPC	-	99	85	74/25	59/26	27 ± 18 m/o	27 ± 17 m/o	Scalp acupuncture	No	PT, swallowing and ST, massage	Three times a week for consecutive ten times with a total of 3 courses, within courses for 15 days pause	VEEG, effective rate
[47]	2015 RGCPC	-	68	68	41/27	43/25	2.3 ± 1.2 y/o	2.1 ± 1.3 y/o	Body acupuncture	No	ST	Five times a week for 3 months	S-SL
[48]	Spastic CP (2015 RGCPC)	-	65	65	36/29	38/27	2.4 ± 1.4 y/o	2.2 ± 1.5 y/o	Body acupuncture	No	PT, OT, massage	Five times a week for 3 months	GMFM-88, MAS
[49]	Meet CP diagnosis	-	30	30	15/15	24/6	28.7 ± 13.8 m/o	35.0 ± 15.1 m/o	Body acupuncture	No	PT	Body acupoint for seven times a week for 3 months; scalp acupoint once every other day for 3 months	Effective rate
[50]	2006 GCPC	-	40	40	22/18	25/15	4 ± 1 y/o	4 ± 1 y/o	Body acupuncture	No	PT, Intelligence motor training	Seven times a week for 20 times as a course for 3 courses, within courses for 3–5 days pause	GMFM-88, MAS
[51]	2006 GCPC	-	100	100	69/31	83/17	1~3 y/o:66; 3~7 y/o:34	1~3 y/o:72; 3~7 y/o:28	Body acupuncture	No	PT, OT, ST	Once every other day for consecutive 20 days with a total of 3 courses, within courses for 15 days pause	GMFM, skull CT/MRI
[52]	2006 GCPC	-	60	60	37/23	39/21	3.26 ± 2.05 y/o	3.51 ± 1.83 y/o	Body acupuncture	No	PT, OT, ST	Seven times a week for 3 months	MAS, GMFM, effective rate
[53]	Spastic CP diagnosis (2004 GCPC)	-	32	30	20/12	17/13	22.3 ± 11.6 m/o	24.6 ± 13.5 m/o	Body acupuncture	Yes	PT, Hyperbaric oxygen therapy	Seven times a week for 60 times	MAS, GMFM
[54]	1988 GCPC	-	40	40	21/19	20/20	5.15 ± 2.78 y/o	6.04 ± 2.37 y/o	Scalp acupuncture	No	PT	Five times a week for 30 days as a course for 3 courses	GMFM, WeeFIM, effective rate
[23]	Spastic CP with type of hemiplegia (2015 RGCPC)	5 (8.6%)	28	30	16/12	17/13	9.89 ± 2.07 y/o	9.37 ± 2.51 y/o	Body acupuncture	Yes	OT	Five days per week for the first two weeks, three days per week for the next two weeks, two days per week for another two weeks, and one day per week for the last two weeks.	NCCPC-R, Squeeze dynamometry, MAS, MMT
[55]	Spastic type	-	39	39	23/16	23/16	37 ± 2 m/o	39 ± 2 m/o	Body acupuncture	No	MyoTrac biostimulation therapy	Five times a week for 6 months	MAS, AROM
[56]	Spastic CP (2015 RGCPC)	-	30	30	18/12	16/14	2.7 ± 0.6 y/o	2.5 ± 0.7 y/o	Scalp acupuncture	No	PT	Five times a week for 6 months	GMFM-88, BBS
[57]	GCPC	-	30	30	16/14	19/11	4.3 y/o	4.1 y/o	Scalp acupuncture	No	PT, balance training	Seven times a week for 90 times	BBS, ADL
[58]	GCPC	-	10	10	4/6	5/5	12.44 ± 6.224 y/o	11.02 ± 7.985	Body/Scalp acupuncture	No	PT	Eight weeks	GMFM
[59]	Spastic type	-	32	31	21/11	23/8	78 ± 32.04 m/o	81.6 ± 37.8 m/o	Scalp acupuncture	No	PT	Six times a week for 60 times	ADL, effective rate
[60]	GCPC	-	33	33	22/11	21/12	4.8 ± 4.0 y/o	6.1 ± 2.89 y/o	Body/Scalp acupuncture	No	PT, massage	Three times a week for 3 months	GMFM
[61]	Spastic CP(2006 GCPC)	-	31	30	19/12	18/12	32.5 ± 1.2 m/o	33.2 ± 1.4 m/o	Scalp acupuncture	No	PT	Five times a week for consecutive 30 days with a total of 3 courses	MAS, GMFM, effective rate

Abbreviations: Rehabilitation Guidelines for Cerebral Palsy in China (RGCPC), Guidelines for Cerebral Palsy in China (GCPC), speech training (ST), non-communicating children’s pain checklist-revised (NCCPC-R), Modified Ashworth Scales (MAS), manual muscle testing (MMT), Gesell developmental diagnostic scale (GESELL), active range of movement (AROM), Berg Balance Scale (BBS), gross motor function measure (GMFM), surface electromyography (sEMG), sign–significate relations for language comprehension and language expression (S-SL), Functional Independence Measure (WeeFIM).

## Data Availability

The data that support the findings of this study are available from the corresponding author upon request.

## Supplementary Materials

The supporting information can be downloaded at https://www.mdpi.com/article/10.3390/healthcare12171780/s1. Figure S1. Trial sequential analysis (TSA) of the Investigator Global Scale of acupuncture for cerebral palsy, which was evaluated by the proportion of subjects with prominent improvements. The effect seemed consistently significant in TSA with appropriate participant number. Table S1. Detailed search strategy employed in this study. Table S2. Acupoints in each selected study.
