# Supplementary material for: Acupuncture and Acupoints for Managing Pediatric Cerebral Palsy: A Meta-Analysis of Randomized Controlled Trials
Source: Healthcare (Basel). 2024 Sep 5;12(17):1780. doi: 10.3390/healthcare12171780 (PMC11395486; doi:10.3390/healthcare12171780)
Supplement: Supplementary file 1 [file healthcare-12-01780-s001.zip › Supplementary Table S1. Detailed search strategy emplyed in this study..pdf]

Supplementary Table S1. Detailed search strategy emplyed in this study.

a. Search strategy used in PubMed

#1	"Cerebral Palsy"[Mesh]	23,550
#2	"Acupuncture"[Mesh]	2,033
#3	"Acupuncture Therapy"[Mesh]	28,967
#4	"Acupuncture, Ear"[Mesh]	501
#5	"Acupuncture Points"[Mesh]	7,931
#6	"scalp acupuncture"	308
#7	"randomized controlled trial"	637,617
#8	animal	7,535,558
#9	#1 AND (#2 OR #3 OR #4 OR #5 OR #6) AND #7 NOT #8	53

b. Search strategy used in Cochrane

#1	"Cerebral Palsy"[Mesh]	2,124
#2	"Acupuncture"[Mesh]	713
#3	"Acupuncture Therapy"[Mesh]	6,405
#4	"Acupuncture, Ear"[Mesh]	241
#5	"Acupuncture Points"[Mesh]	2,500
#6	"scalp acupuncture"	358
#7	"randomized controlled trial"	641,573
#8	animal	26,910
#9	#1 AND (#2 OR #3 OR #4 OR #5 OR #6) AND #7 NOT #8	21

c. Search strategy used in EMBASE

#1	cerebral palsy*'	52,703
#2	'acupuncture'	64,304
#3	'acupuncture therapy'	2,430
#4	'acupuncture, ear'	40
#5	'acupuncture points'	2,323
#6	'scalp acupuncture'	472
#7	'randomized controlled trial'	1,038,235
#8	animal	6,972,116
#9	#1 AND (#2 OR #3 OR #4 OR #5 OR #6) AND #7 NOT #8	97

d. Search strategy used in SCOPUS

#1	TITLE-ABS-KEY ("cerebral palsy*"	47,453
#2	TITLE-ABS-KEY ("acupuncture")	55,479

#3	TITLE-ABS-KEY ("acupuncture therapy")	18,873
#4	TITLE-ABS-KEY ("acupuncture, ear")	465
#5	TITLE-ABS-KEY ("acupuncture points")	10,359
#6	TITLE-ABS-KEY ("scalp acupuncture")	453
#7	TITLE-ABS-KEY ("randomized controlled trial")	971,601
#8	TITLE-ABS-KEY (animal)	8,203,680
#9	#1 AND #2 OR #3 OR #4 OR #5 OR #6 AND #7 AND NOT #8	80

e. Search strategy used in CNKI

#1	Nǎo tān + Xiǎo ér nǎo tān + Nǎo xìng tān huàn + Jìng luán xìng nǎo tān + Ér tóng nǎo tān + Xiǎo ér nǎo xìng tān huàn	73,295
#2	Zhēn jiǔ + Zhēn jiǔ fǎ + Zhēn jiǔ fāng fǎ + Zhēn jiǔ liáo fǎ + Zhēn jiǔ zhì liáo + Zhēn zhì fǎ + Zhēn cì liáo fǎ + Tóu zhēn + Tóu pí zhēn + Tóu zhēn liáo fǎ + Ěr zhēn + Ěr zhēn liáo fǎ + Ěr zhēn zhì liáo + Tǐ zhēn	167,095
#3	Lín chuáng shí yàn + Lín chuáng shì yàn + Suí jī duì zhào shuāng máng shì yàn + Suí jī duì zhào shuāng máng shí yàn + Suí jī duì zhào shì yàn shè jì + Suí jī duì zhào shí yàn shè jì + Suí jī shuāng máng duì zhào shì yàn + Suí jī shuāng máng duì zhào shí yàn + Suí jī duì zhào + Suí jī duì zhào zhì liáo + Suí jī shuāng máng duì zhào + Duì zhào shí yàn + Duì zhào shì yàn + Lín chuáng guān chá + Liáo xiào guān chá + Lín chuáng yán jiū	2,296,576
#4	Dòng wù	2,581,804
#5	#1 AND #2 AND #3 NOT #4	105
