# Supplementary material for: Acupuncture and Acupoints for Managing Pediatric Cerebral Palsy: A Meta-Analysis of Randomized Controlled Trials
Source: Healthcare (Basel). 2024 Sep 5;12(17):1780. doi: 10.3390/healthcare12171780 (PMC11395486; doi:10.3390/healthcare12171780)
Supplement: Supplementary file 1 [file healthcare-12-01780-s001.zip › Supplementary Table S2.pdf]

Supplementary Table S2. Acupoints in each selected study

Chen et al. (2021)	EX-HN1, Jhih san jhen, Nao san jhen, Nie san jhen, DU14, DU12, DU4, BL23, ST36, SP6, KL3(plus following acupoints according to the individuals symptoms: BL18, BL23, BL15, BL17, BL20, BL21, ST40, SP10, RN12, RN6, LR3)
Zhang et al. (2019)	GV 20, EX-HN1, MS6, EX-B2, BL40, GB39, ST36, LI4(plus following acupoints according to the individuals symptoms:BL18, BL23, BL15, BL20, SP10, ST40, LI15, LI11, LI10, SJ5, LU10, EX-UE9, GB30, GB34, ST40, ST41, SP6, BL60, LR3
Zhao et al. (2017)	GV20, GV16, GV12, GV9, GV8, GV4, GV3, BL20, BL23, ST36, SP6
Li et al. (2017)	GV24, GB13, EX-HN1, combined with MS6, MSJ6, MSJ7, MSJ9, MS14, Nie san jhen, BL9
Du et al.(2017)	BL20, ST34, SP6, LR3 (plus following acupoints according to the individuals symptoms:BL18, BL20, BL23, HT6, HT7, ST32, ST34, ST36, LR3, LR4, LR6, SP4, SP6, SP10,
Du et al.(2016)	SI9, LI15, LI14, SJ14, LI10, ST34, GB31, GB34, BL37 BL60, , BL20, BL18, BL24, BL33
Zhang, N. X et al. (2014)	EX-B2, LI15, LI11, LI4, GB34, SP9, GB39, ST36, SP6,BL57, LR3, KI3, HT7, DU20, Jhih san jhen, EX-HN1, Nao san jhen, Nie san jhen, MS6
Zhang, N et al. (2014)	EX-HN1, Nao san jhen, Jhih san jhen, Nie san jhen, jùsānzhēn, xīsānzhēn, ST36, SP6, LR3, ST35, ST34, SP10, BL56, BL57 (plus following acupoints according to the individuals symptoms: KI3, ST41, GB39, BL60, GB31, GB34, EX-LE10)
Liu et al. (2013)	DU2, DU3, DU4, DU5, DU6, DU7, DU8, DU9. DU10, DU11, DU12, DU13, DU14, BL23, KI3, GB34, ST36, SP6, DU20, DU21,DU24, DU17, EX-HN1
Wang et al. (2011)	MS14
Zhou et al. (2010)	EX-B2
Ji et al. (2008)	DU20, EX-HN1, MS6, MSJ11, MSJ2, MSJ3, MSJ9, MSJ6, MSJ7, MSJ9, MSJ3
Zhao et al. (2022)	EX-UE9, LI4, SI3, LI10, LI11
Zhang et al. (2020)	EX-HN1, Jhih san jhen, Nao san jhen, Nie san jhen, Shou san jhen, Shou jhih jhen, DU17, DU24, GB13, GB19, LI11, SJ5, LI4, PC6, PC8, HT7
Luo et al. (2020)	MS6, MSJ2, SJ9, MSJ11
Zhang et al. (2012)	DU20, DU21, DU17, DU16, BL10
Chen et al. (2017)	DU20, EX-HN1, Jhih san jhen, Nie san jhen, MS6, MSJ6, EX-B2
Lee et al. (2009)	EX-HN1, Jhih san jhen, Nao san jhen, Nie san jhen, DU17, DU24, GB13, GB19
Lee et al. (2013)	MS6, MSJ9, DU20, EX-HN1, MSJ6, MSJ7, (plus following acupoints according to the individuals symptoms: LI15, LI11, SI3, LI4, SJ3, SJ5, ST32, ST34, SP10, ST36, ST40, GB34, GB39, KI3, LR3)
Yang et al. (2016)	DU20, EX-HN1, DU24, GB13, MS6, MS14

* Jhih san jhen : DU24,GB13

* Nao san jhen : DU17,GB19

* Nie san jhen : GB6,GB8,GB9

* jùsānzhēn : ST36,SP6,LR3

* xīsānzhēn : EX-LE4,EX-LE5,GB34
