# Supplementary figures and images for: Acupuncture and Acupoints for Managing Pediatric Cerebral Palsy: A Meta-Analysis of Randomized Controlled Trials
Source: Healthcare (Basel). 2024 Sep 5;12(17):1780. doi: 10.3390/healthcare12171780 (PMC11395486; doi:10.3390/healthcare12171780)

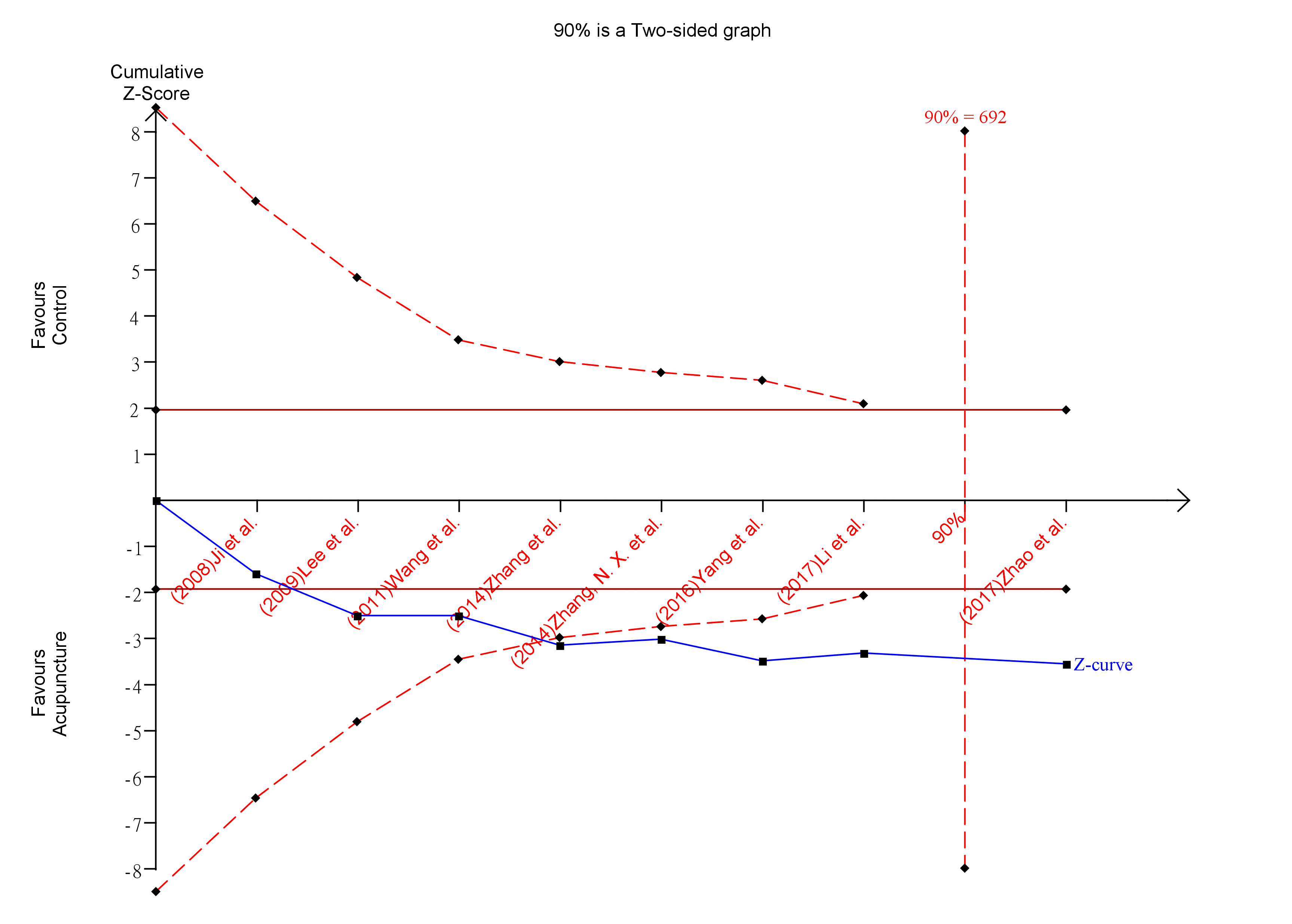

Supplement: Supplementary file 1 [file healthcare-12-01780-s001.zip › Supplementary Figure S1.png]
